# The Emerging Boon of Information and Communication Technology in Multidisciplinary Cancer Care: A Force Multiplier With a Human Touch

**DOI:** 10.7759/cureus.33665

**Published:** 2023-01-11

**Authors:** Srinivasan Vijayakumar, Frances B Lancaster, Mary R Nittala, William N Duggar

**Affiliations:** 1 Radiation Oncology, University of Mississippi Medical Center, Jackson, USA

**Keywords:** multidisciplinary tumor board, quality of life, cancer care, total package time, information and communication technology

## Abstract

Cancer care (CC) is incredibly complex and requires the coordination of multiple disciplines for optimal outcomes. Historically, this has been accomplished with multidisciplinary tumor boards (MDTBs), but the benefits, while perhaps intuitive, have not always been demonstrated with sufficient research robustness and validity. We hypothesize that this difficulty in demonstrating the benefit of MDTBs may be related to a delay in decision-making and operationalizing those decisions. The history and value of MDTBs are presented as well as their weaknesses and limited demonstration of improved outcomes. A major weakness highlighted by the challenges of MDTBs is the concept of total package time (TPT) (rather, the inability to keep it as short as possible); any significant delays in CC for any discipline may have a deleterious impact on any given patient’s care outcome. Drawing on our own experience with utilizing information and communication technology (ICT) during an effort to apply accountability theory to improve specifically radiation therapy package time (RTPT), we argue that similar principles will be applicable in the improvement of not only the TPT which relies on multiple disciplines, but other factors of CC as well, such as coordination. Experience with improvement in RTPT is discussed and the underlying theory is demonstrated as a sound methodology to apply beyond RTPT to TPT involving coordination of multiple disciplines and stands to lead to the full realization of the benefits of the multidisciplinary approach. The complexity of cancer means that real solutions to optimal outcomes are also, by nature, complex, but here simple accountability theory is demonstrated that may unlock the next phase of multidisciplinary coordination. In this work, we argue that the benefits of the MDTB format can be fully realized with the addition of ICT, a technological breakthrough in the past two decades, while not forgetting about continued human factors.

## Introduction and background

Cancer is a major cause of mortality, morbidity, loss of quality of life, and interruption in the life of patients and their families as well as a major drain on the gross domestic product of nations [1]. Furthermore, over 60-80% of the cost of cancer care (CC) happens in the last months and weeks of the patients’ demise from uncontrolled cancer. The loss of income to the patients and their families during the CC, the cost of CC to the family, and if the patient ends up with disabilities and unable to work or dies due to cancer, the subsequent loss of income for the family can all add to the economic sufferings for the family [2] and the necessity to address the financial toxicity in CC [3]. So, every effort should be made to improve the control of cancer with a goal to cure as high a percentage of patients as possible, because the reduction in human suffering is immeasurable; in addition, rehabilitation following cancer treatment can have many economic benefits to the families, communities, societies, and nations [4].

Whereas scientists often focus on treatment breakthroughs by improving surgical, radiotherapy (RT), chemotherapy, and other systemic therapy interventions as well as early diagnosis, prevention, and vaccine developments, it is often forgotten that attention to almost mundane steps such as coordination of cancer care (CoCC) in terms of quick diagnosis after a symptom develops, rapid cancer staging, and avoiding delays in starting and completing treatments can all make huge differences in improving survivals and in turn decrease the human sufferings with added benefits of economic benefits to the patients, their families, societies, and nations.

In this paper, we will outline how information technology (IT)’s and more specifically, information and communication technology (ICT)’s growth and breakthroughs in the past two decades can be taken advantage of to improve many of the steps that require a CoCC (Table 1).

**Table 1:** Steps that need coordination to provide the optimal cancer care; what this paper will address and what it will not

Steps in the cancer diagnosis and treatments - cancer care		What will and will not be addressed in this paper in terms of the use of information and communication technology in the coordination of cancer care to improve outcomes
1	Getting the patient to the right provider for the potential cancer diagnosis in the shortest time since the starting of symptoms	No
2	Completion of biopsy and correct pathological diagnosis of cancer including modern molecular pathology and next-generation sequencing as indicated	No
3	Completion of the cancer staging workup	Partially yes
4	Assembling the right group of multidisciplinary teams relevant to the site of cancer to make treatment recommendations	Yes
5	A quick turn-around time to get a multidisciplinary tumor board arranged to come up with a treatment plan	Yes
6	Quick completion of the planning process of the sequencing of treatments and starting of the treatment	Yes
7	Supportive steps needed for the patient and family to complete all the treatments in the ideal time span planned	Yes

This article intends to show that (a) a delay in the quick diagnosis of cancer, (b) a delay in the rapid scheduling of investigative workup to clinch the right diagnosis and staging of cancer, (c) a delay in gathering the right team of CC specialists together to provide the right treatment for the right patient at the right time and finally, and (d) completing the planned treatment course within the planned period all can be improved by judicial use of ICT with a human touch [5].

## Review

Methods

Search Databases and Search Strategy

This is a narrative review where databases such as MEDLINE/PubMed, and Google Scholar were used for the literature search. These databases, within the time period of 2010 and 2022, were screened by our research team from November 12, 2022, to December 18, 2022, with individual medical subject heading keywords and key term combinations including “multidisciplinary tumor boards," “total package time (TPT),” “radiation therapy package time (RTPT),” “cancer care,” “information technology (IT),” “information and communication technology (ICT)."

Inclusion/Exclusion Criteria

Studies only in the English language from 2010 to 2022 were included, with no other specific filters being used. Commentaries, letters to the editor, and unpublished reports were excluded.

Multidisciplinary approach to cancer care

CC is complex; it is complicated, prolonged (sometimes for months to even a year or longer), and in the majority of circumstances involves many specialists [6]. Multidisciplinary teams (MDTs) have been utilized in CC for over 50 years [7]. The use of MDTs in treating patients with cancer is both necessary and complex and over time evolved further into the common use of multidisciplinary tumor boards (MDTBs) which have shown many advantages and disadvantages as shown in Table 2 [8].

**Table 2:** Advantages and disadvantages of multidisciplinary tumor boards MDTBs: multidisciplinary tumor boards This table is adapted from Berardi et al. [8], and permission was obtained from licensed content publisher Dove Medical Press Ltd.

Pros		Cons	
1	Adherence to clinical guidelines	1	Not a significant impact on outcomes
2	Mechanism for review of the quality of professional care	2	High time expenditure and economic cost
3	Management of rare tumors and/or with clinical guidelines lacking	3	Low quality of information and lack of fundamental reports presented to MDTBs
4	Improvement of ability to reach decisions, quality of information presentation, and quality of teamwork	4	Excessive not strictly clinical information might lead to contrasting opinions
5	Improvement in patient outcomes	5	Legal issues related to responsibilities in confidentiality and anonymity of every patient presented to MDTBs
6	Change in diagnostic or treatment plans	6	Accessibility to national networks and MDTBs, owing to geographic origin and socioeconomic conditions
7	Improvement in follow-up accuracy	7	Risk of treatment delays
8	Improvement in clinical trial screening and patient recruitment		

The lack of demonstrable survival/ disease control outcome improvement is likely to be real, due to a prolongation of TPT. This is our hypothesis, and that led to the current communication.

A multidisciplinary approach is beneficial for the patient because it enables better long-term follow-up with the primary care physician after treatment [6,9]. In addition, a multidisciplinary approach is associated with some improved quality of care measures [10], and adherence to guidelines [8]. Although there are sparse data showing outcome improvements with MDTs, studies are starting to demonstrate the advantages of MDTBs in recent years. A retrospective study showed that for a substantial proportion of patients there were no decided treatment plans before a thoracic MDTB and in close to 50% of those who had a previous plan of management, a change in plan resulted from the MDTB discussion [11]. Another recent assessment of the benefits of molecular MDTB showed similar advantages in terms of changes in management plans or a need for a new test [12]. The above two studies justify continuing with the MDTBs in helping make decisions for cancer patients. They also point out a dire need to construct new initiatives to (a) assess the outcome benefits of MDTBs; (b) document TPTs and design new ways to shorten the TPT and make MDTBs more timely in decision making and being efficient; (c) take advantage of the new communication information technology to make those two steps possible [5,13]. The potential benefits of using telemedicine methods and artificial intelligence interventions should not be neglected.

MDTBs help brings specialists together to confer on the best patient care in light of the ever-changing evidence and updated guidelines. Surgeons, radiation oncologists, medical oncologists, radiologists, pathologists, internists, and in some cases, nurses, make up the core team that comprises an MDTB [8].

The staging of cancer is an invaluable step in the CC since the cancer stage determines the intensity and types of treatment interventions, as well as the predicted prognosis [14]. However, steps and investigations to complete the staging can cause a delay in starting appropriate treatment [15]. Moreover, the specialists and staff must communicate effectively with each other and patients/families to adequately provide resources, make appointments, and coordinate the CC [16]. Lack of effective communication amongst healthcare providers is a prominent cause of adverse events in CC, with many of the problems stemming from failure to transmit the information or the sending of inaccurate information [6].

**Figure 1:**
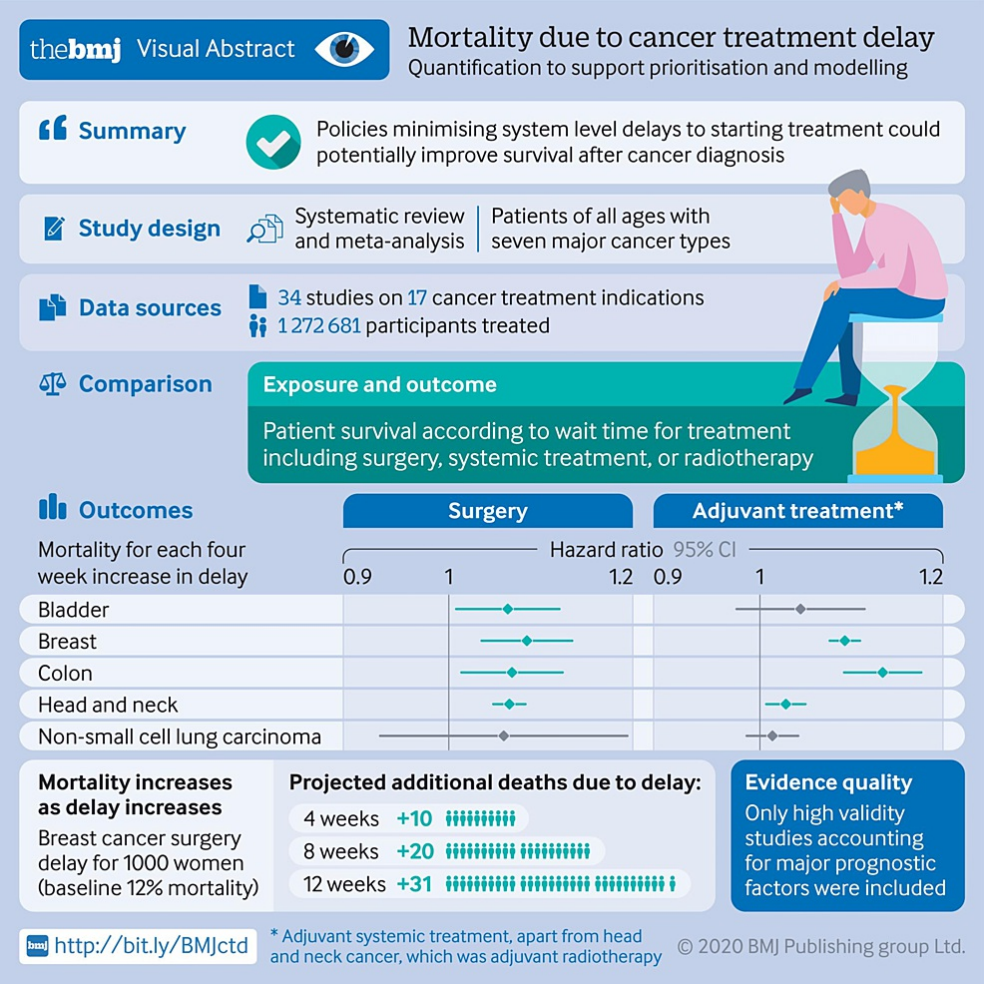
Mortality from delay in treatments in cancer care CI: confidence interval, %: percentage This image is reproduced from Hanna et al. [17], and permission was obtained from the license content publisher BMJ Publishing Group Ltd.

MDTBs also require the scheduling of multiple providers, which can delay treatment [8]. A delay in CC is often associated with a delay in the cure, adequate palliation, or loss of quality of life. In a meta-analysis examining 34 studies, every 28-day delay between diagnosis of cancer and surgical intervention was correlated with a relative increase of 6-8% in all-cause mortality; a 9-23% mortality increase was noted in delay for radiotherapy treatment as opposed to surgery. However, confounding variables (such as failure to separate results based on different cancer stages) and limited use of the 34 total studies limit the effective extrapolation of results seen (Figure 1) [17].

Regardless, important insights can be garnered from the data gathered in the study, and the information regarding mortality due to cancer delay is included in the pictorial form above. The coronavirus disease 2019 (COVID-19) pandemic roused deferrals in elective RT and surgeries in several regions, including British Columbia [18]. Concern over delays in treatment due to COVID-19 has recently roused interest in determining how pandemics can or other national crises can affect survival outcomes and treatment success among patients with cancer [17]. Avoiding delay requires a team-based approach that can efficiently prioritize the care of the patient and make collaborative decisions quickly [19]. Effective use of digital information technology-based interventions can accomplish these goals, and this paper will demonstrate how that is possible by synthesizing data and evidence from multi-disciplinary sources.

Information and communication technology, and physician-patient communication

ICT implementations and afforded opportunities are still emerging, but it has already been shown to be associated with improved access to care as well as improved communication and trust among patients with their physicians [20]. With the emotional nature of a cancer diagnosis, physician-to-patient communication must face and overcome additional barriers such as the patient’s increased cognitive demand. Using ICT can help overcome some barriers by providing an avenue for patients to ask questions after allowing time for information to sink in [20]. In addition, ICT can provide more discrete and succinct answers to questions that may have been unsatisfactorily answered. On the other hand, ICT can take time away from other physically present patients and can create extra work for providers who may not appreciate added benefits [20].

There is evidence that many patients respond positively to using ICT as an adjunct to in-person discussion with a physician [20]. Patient-provider communication is critical in a cancer diagnosis [21]. Patients with breast cancer in one study reported an increased ability to cope with their illnesses and a greater rapport with their physicians [20]. ICT may improve the quality of care, increase data sharing, and even facilitate difficult conversations that will clear up patient confusion [22]. ICT can also foster shared decision-making, which can empower patients to make the best decisions for themselves [20]. Opinions among providers and patients may differ regarding the use of ICT, one study found patients reported empowerment from increased access to online resources, while physicians reported concerns about potential patient confusion and distress [23]. Patients report increased trust in medical professionals when they encourage seeking information using online resources [24].

The importance of total package time

In light of the limitations of MDTBs, operational tools must be used to minimize the aforementioned delay and therefore TPT, defined as the amount of time between surgery and the last day of radiation [25]. A shorter TPT is important for patient outcomes, as a longer TPT has been linked with worse survival outcomes [26]. ICT can be utilized to help with data collection in the context of multi-disciplinary CC [27]. ICT allows for efficient use of resources (such as patient imaging) and effective communication among team members separated geographically. Both technological and human tools are necessary to achieve a decrease in TPT, as the use of ICT alone has not been consistently linked with improved care [27].

There are several benefits to adopting ICT in medical care. ICT has been able to improve the ability to enroll patients with cancer in clinical trials [28]. Other forms of ICT increase a practice’s accountability by comparing the outcomes of patients to national benchmarks [27]. However, the adoption of new forms of technology is often met by barriers among healthcare providers and staff, including inadequate product reliability, difficulty integrating into practice, and resistance to ideological reorganization [29]. The authors' own experience with SmartClinic (Elekta, Stockholm, Sweden) has demonstrated the success of using ICT to reduce RTPT. If the challenges of adopting a new system can be faced and confronted, the improved accountability seen with SmartClinic and other forms of ICT will lead to improved patient outcomes.

Application of accountability theory to the clinic

**Figure 2:**
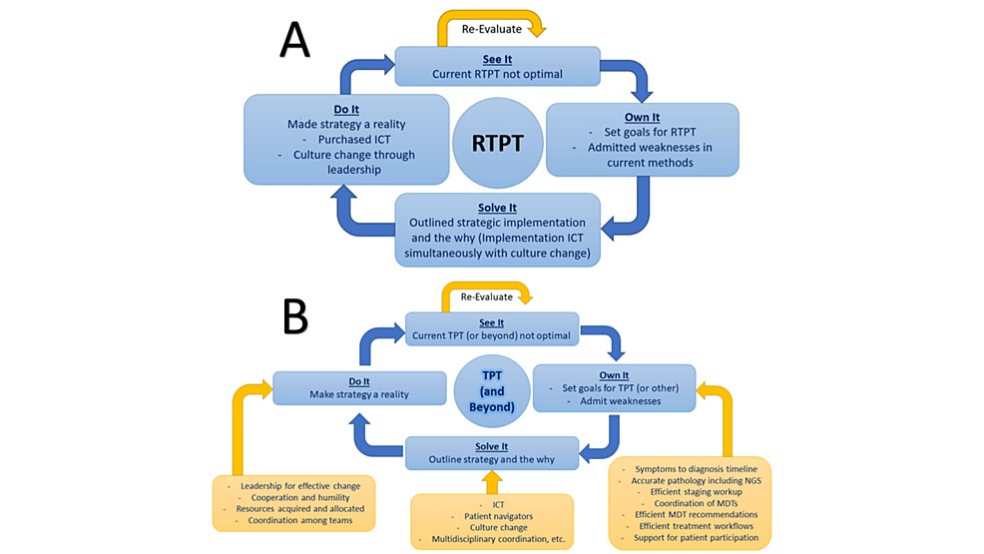
Application of accountability theory to the clinic RTPT: radiation therapy total package time, TPT: total package time, ICT: information and communication technology, MDT: multidisciplinary team A) The application of "The Oz Principle" accountability theory to the improvement of RTPT. B) Demonstration of how the same accountability theory could be applied to other concepts, such as overall TPT and suggestions for considerations for each step in the practical application This image is a simplified depiction from the authors' of this paper

In the book, The Oz Principle, a popular strategy for building healthy accountability is outlined by the terms: “See it, Own it, Solve it, Do it”. The idea is that a problem is identified and an opportunity for improvement is identified. In this case, this “See it” step was the acknowledgment of the need for improvement to RTPT. “Own it” meant defining goals regarding what improvement should look like. The “Solve it” step involved creating a specific strategy to make it happen and defining the need to put the strategy into action. Finally, “Do it” meant not only acquiring the necessary resources (i.e. ICT) and configuring them to work properly in the clinical workflow, but also leading a culture change within the team to not only adopt the new related workflow but to submit to the healthy accountability in a manner that incited motivation rather than any need for discipline or negative feelings (Figure 2) [30].

The following paragraphs detail these efforts to put this accountability strategy into action.

Hypothesis

Delays in the implementation of patient treatment plans are a problem that creates an increase in TPT. We believe that the use of ICT, such as SmartClinic, and human interventions such as patient navigators can be used to decrease TPT. We also aim to specifically address a reduction in the RTPT.

Importance of multidisciplinary tumor board within radiation oncology 

MDTBs are a valuable part of cancer care, but we want to offer suggestions of how they can be improved. Operational tools are needed to make the promises of MDTBs fully realized because they can improve the efficiency and efficacy of MDTB, streamlining information for all involved in patient care. However, we will need a human component as well, and we would recommend patient navigators. Patient navigators will be needed to help communicate with the patient after the MDTB, and navigators also offer a more personalized approach. We also see a need to improve interdisciplinary care coordination and increase patient involvement; by improving both of these, we will increase patient autonomy and thorough, holistic care. By designing clinical trials, we can test these hypotheses. Radiation oncology-specific tumor boards are needed for optimal care and optimal TPT for our patients. Disciplines that participate include physicians, physical therapists, dosimetrists, nurses, patient navigators, therapists, clinical research personnel, and trainees for each field.

**Figure 3:**
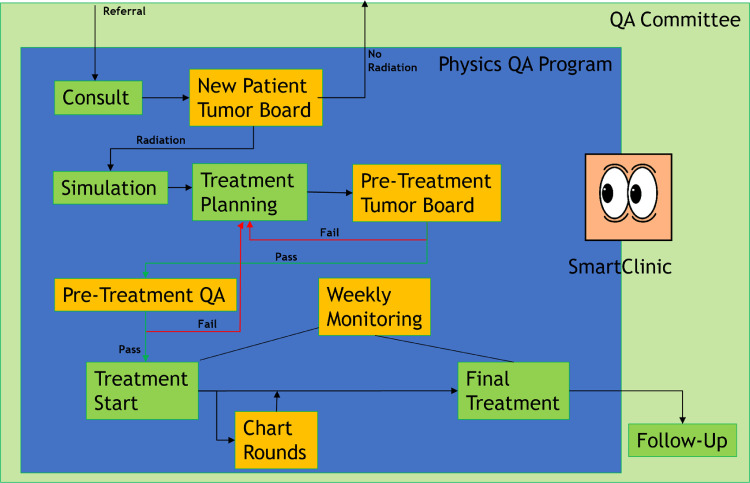
An illustration of steps taken in a Radiation Medicine department The steps depicted are taken to expedite the entire treatment coordination process and decrease the total package time, and radiation therapy total package time using information and communication technology with a human touch. QA: quality assurance This image is reproduced from Duggar et al. [5], and permission was obtained from licensed content publisher Elsevier

The radiation oncology-specific tumor board will need to review three case scenarios at varying intervals: new cases, pre-treatment peer review cases, and chart rounds. Below is an example of a schematic of an intradepartmental tumor board (Figure 3) [5].

It illustrates the importance of following a systematic, orderly sequence in reviewing cases so that care is both efficient and thorough. ICT can be utilized in an MDTB. In addition, ICT can be used to improve TPT, and more specifically, RTPT. The University of Mississippi Medical Center (UMMC) has experience in using ICT to reduce RTPT. Strategic intervention to improve package time needs ICT, but it also needs less tangible, more personal components, such as accountability and guidance of a team [5]. When a need was sensed to streamline care and reduce RTPT at UMMC, several implementations were made and then evaluated for efficacy. A new treatment planning system (TPS) was implemented first followed by the use of ICT (SmartClinic) combined with important human factors such as accountability, motivation, and leadership to yield desired results of improved RTPT, the human factors of this implementation cannot be ignored in evaluation [5]. The TPS was changed from Pinnacle version 16.2 (Philips, UK) to RayStation version 9A (Raysearch Laboratories, Stockholm, Sweden).

The ICT consisted of a method of patient tracking, healthy accountability, and resource allocation for implementation (software, faculty, or workflow changes) was also initiated to make the needed changes to streamline the intervention. The SmartClinic system was utilized to automate patient timeline tracking at UMMC, using information that was uploaded from Mosaiq. A SmartBoard was visible on a screen in the conference room area and was reviewed each morning during the daily huddle. The SmartBoards were also visible from any computer, remote, or in the clinic. Healthy accountability was established by departmental chair oversight and leadership, color-coded due dates for workflow steps for each patient, and open communication between the departmental chair and other staff regarding implementation, expectations, and barriers to use. The departmental response to the new interventions was met with overwhelmingly positive sentiment [5].

When only the TPS was updated, there was no significant improvement in RTPT. However, when an updated TPS was used in combination with automated patient management and accountability system, there was a significant improvement in RTPT. For 112 patients with head and neck (HN) cancer, there was an overall reduction of RTPT of 22.85 days (p=0.002) and a reduction in a median of RTPT of 14 days (p=0.006) when analyzing and reviewing the average time difference between patient populations before and after these interventions were made, over two years. In conclusion, the combination of improved ICT and human accountability and leadership led to shortened RTPT [5]. This experience demonstrates that ICT can address challenges that currently exist within the complexity of the use of MDT and MDTB and when combined with human factors, can impact treatment efficiency and ultimately outcomes.

Importance of research in avoiding treatment delays - human and non-human (artificial intelligence) interventions

As discussed above, delays in cancer treatment are linked to an increase in all-cause mortality. The meta-analysis study demonstrates the importance of minimizing the waiting time for patients with cancer [17]. For example, poor survival and late-stage presentation were associated with a delay of at least three months in a systematic review of 87 breast cancer studies [31]. However, there is a need for more research to better specify the effects of delay in treatment on time to radiotherapy [18]. A delay in treatment would be naturally assumed to be associated with a worse outcome, but there is little data on the delay versus outcome relationship [32].

During the COVID-19 pandemic, many guidelines encouraged patients with a diagnosis of cancer to delay elective surgery, with little understanding of what kind of an impact that might have on patient morbidity and mortality [32]. Cancer surgery centers were especially vulnerable to lockdowns during the COVID-19 pandemic, with millions of cancellations in the first 12 weeks of the first COVID-19 wave [33]. One study examined retrospective evidence on the effect of a delay of three months or more after diagnosis of colorectal cancer, and the authors found a negative effect on the overall survival and disease-free interval, particularly when surgery was delayed beyond three months. However, the authors recognized that more data and research need to be done to better understand the relationship between delay in treatment and patient outcome [32]. With the progress being made in the use of artificial intelligence (AI), we can anticipate the automation of monitoring of many steps in the diagnosis, treatment decision making and treatment operationalizing processes of CC (see Table 1). These automated monitoring and alerting of humans by AI processes can help shorten the TPT successfully in the future, yet still, reap the benefits of MDTBs [34].

## Conclusions

For better and worse, MDTBs have become a hallmark of modern CC due to the complexity of providing optimal and efficacious treatment. The final treatment decision does not rely on the expertise of only one individual, but instead on collective expertise from many disciplines. The benefits of this approach are not without cost nor as comprehensive as one may wish, as clinical efficiency and TPT can be largely affected, and collective expertise is still limited to the skills, knowledge, and egos of the individuals who make up the MDTB. We currently stand upon a precipice of opportunity to further advance the clinical impact of our collective effort at MDTBs through the strategic adoption of various technologies discussed above (i.e. ITC, AI, etc.). One example was even shared of the use of ICT to improve TPT (especially RTPT) without degradation of the MDTB format. Further implementation would achieve similar goals while also increasing patient involvement which arguably is specific expertise currently left out of MDTB decisions to some degree. Additionally, successful intervention with ICT/AI would improve coordination among disciplines beyond the MDTB and perhaps even result in infrastructure for efficient clinical trials and protocols to test these hypotheses.

In any given cancer patient’s care, innumerable professionals are involved with the number increasing all the time. Based on published data, delays from diagnosis to treatment will impact clinical outcomes for these patients, especially regarding disease control and survival. Despite this being intuitive, the required coordination of care and the necessity for MDTBs to minimize delays can be quite difficult. Incorporation of ICT into patient and care pathways stands as a potential next step to optimize care for more and more patients. The successful strategic efforts mentioned above in improving RTPT with SmartClinic should not only be reproducible in other settings but are of paramount importance to improve not only TPT but also CC management in general.

## References

[1] Yabroff KR, Mariotto A, Tangka F (2021). Annual report to the nation on the status of cancer, Part 2: Patient Economic Burden Associated With Cancer Care. J Natl Cancer Inst.

[2] Angioli R, Capriglione S, Aloisi A (2015). Economic impact among family caregivers of patients with advanced ovarian cancer. Int J Gynecol Cancer.

[3] Smith GL, Banegas MP, Acquati C (2022). Navigating financial toxicity in patients with cancer: a multidisciplinary management approach. CA Cancer J Clin.

[4] Silver JK, Baima J, Newman R, Galantino ML, Shockney LD (2013). Cancer rehabilitation may improve function in survivors and decrease the economic burden of cancer to individuals and society. Work.

[5] Duggar WN, Weatherall L, Nittala MR (2023). Strategic reduction of package time in head and neck cancer. Adv Radiat Oncol.

[6] Easley J, Miedema B, Carroll JC, Manca DP, O'Brien MA, Webster F, Grunfeld E (2016). Coordination of cancer care between family physicians and cancer specialists: importance of communication. Can Fam Physician.

[7] Dickhoff C, Dahele M (2019). The multidisciplinary lung cancer team meeting: increasing evidence that it should be considered a medical intervention in its own right. J Thorac Dis.

[8] Berardi R, Morgese F, Rinaldi S, Torniai M, Mentrasti G, Scortichini L, Giampieri R (2020). Benefits and limitations of a multidisciplinary approach in cancer patient management. Cancer Manag Res.

[9] Van Dijk-de Vries AN, Duimel-Peeters IG, Muris JW, Wesseling GJ, Beusmans GH, Vrijhoef HJ (2016). Effectiveness of teamwork in an integrated care setting for patients with COPD: development and testing of a self-evaluation instrument for interprofessional teams. Int J Integr Care.

[10] Reiss-Brennan B, Brunisholz KD, Dredge C (2016). Association of integrated team-based care with health care quality, utilization, and cost. JAMA.

[11] Kreidieh F, Tfayli A (2023). Impact of thoracic multidisciplinary tumor boards on the management of patients with cancer: a retrospective study at the American university of Beirut medical center. Mol Clin Oncol.

[12] Behel V, Noronha V, Choughule A (2022). Impact of molecular tumor board on the clinical management of patients with cancer. JCO Glob Oncol.

[13] Gebbia V, Guarini A, Piazza D (2021). Virtual multidisciplinary tumor boards: a narrative review focused on lung cancer. Pulm Ther.

[14] SEER Training Modules, Purpose of Staging. U. S (2023). SEER Training Modules, Purpose of Staging. U. S National Institutes of Health, National Cancer Institute. https://training.seer.cancer.gov/staging/intro/purpose.html.

[15] Leiro-Fernández V, Mouronte-Roibás C, García-Rodríguez E (2019). Predicting delays in lung cancer diagnosis and staging. Thorac Cancer.

[16] Gorin SS, Haggstrom D, Han PK, Fairfield KM, Krebs P, Clauser SB (2017). Cancer care coordination: a systematic review and meta-analysis of over 30 years of empirical studies. Ann Behav Med.

[17] Hanna TP, King WD, Thibodeau S (2020). Mortality due to cancer treatment delay: systematic review and meta-analysis. BMJ.

[18] Parmar A, Chan KK (2020). Prioritising research into cancer treatment delays. BMJ.

[19] Blackwood O, Deb R (2020). Multidisciplinary team approach in breast cancer care: benefits and challenges. Indian J Pathol Microbiol.

[20] ElKefi S, Asan O (2021). How technology impacts communication between cancer patients and their health care providers: a systematic literature review. Int J Med Inform.

[21] Yu L, Zheng F, Xiong J, Wu X (2021). Relationship of patient-centered communication and cancer risk information avoidance: a social cognitive perspective. Patient Educ Couns.

[22] Ansari N, Wilson CM, Heneghan MB, Supiano K, Mooney K (2022). How technology can improve communication and health outcomes in patients with advanced cancer: an integrative review. Support Care Cancer.

[23] Ochoa-Arnedo C, Flix-Valle A, Casellas-Grau A (2020). An exploratory study in breast cancer of factors involved in the use and communication with health professionals of Internet information. Support Care Cancer.

[24] Bylund CL, Gueguen JA, D'Agostino TA, Li Y, Sonet E (2010). Doctor-patient communication about cancer-related internet information. J Psychosoc Oncol.

[25] Ghanem AI, Schymick M, Bachiri S (2019). The effect of treatment package time in head and neck cancer patients treated with adjuvant radiotherapy and concurrent systemic therapy. World J Otorhinolaryngol Head Neck Surg.

[26] Suzuki H, Tamaki T, Tsuzuki H, Nishio M, Nishikawa D, Beppu S, Hanai N (2020). Association between treatment package time and clinical predictors in oropharyngeal cancer. Medicine (Baltimore).

[27] Janssen A, Robinson T, Brunner M, Harnett P, Museth KE, Shaw T (2018). Multidisciplinary teams and ICT: a qualitative study exploring the use of technology and its impact on multidisciplinary team meetings. BMC Health Serv Res.

[28] von Itzstein MS, Hullings M, Mayo H, Beg MS, Williams EL, Gerber DE (2021). Application of information technology to clinical trial evaluation and enrollment: a review. JAMA Oncol.

[29] Lluch M (2011). Healthcare professionals' organisational barriers to health information technologies-a literature review. Int J Med Inform.

[30] Connors R, Smith T, Hickman C (2004). The Oz principle: getting results through individual and organizational accountability. Penguin.

[31] Nguyen SM, Nguyen QT, Nguyen LM (2021). Delay in the diagnosis and treatment of breast cancer in Vietnam. Cancer Med.

[32] Whittaker TM, Abdelrazek ME, Fitzpatrick AJ, Froud JL, Kelly JR, Williamson JS, Williams GL (2021). Delay to elective colorectal cancer surgery and implications for survival: a systematic review and meta-analysis. Colorectal Dis.

[33] (2021). Effect of COVID-19 pandemic lockdowns on planned cancer surgery for 15 tumour types in 61 countries: an international, prospective, cohort study. Lancet Oncol.

[34] Bhinder B, Gilvary C, Madhukar NS, Elemento O (2021). Artificial intelligence in cancer research and precision medicine. Cancer Discov.

